# Nitrogen Export From a Watershed Subjected to Partial Salvage Logging

**DOI:** 10.1100/tsw.2001.290

**Published:** 2001-11-09

**Authors:** Maria Herrmann, William E. Sharpe, David R. DeWalle, Bryan R. Swistock

**Affiliations:** School of Forest Resources and Environmental Resources Research Institute, The Pennsylvania State University, University Park 16802, USA

**Keywords:** logging, nitrogen, nitrate, stream export, leaching, forest decline

## Abstract

Logging has been shown to induce nitrogen (N) leaching. We hypothesized that logging a watershed that previously exhibited forest decline symptoms would place additional stress on the ecosystem and result in greater N loss, compared to harvesting vigorous forests. We conducted a 10-year (1988 to 1998) assessment of N export from the Baldwin Creek watershed in southwestern Pennsylvania that was partially clearcut to salvage dead and dying northern red oak. N export from the watershed increased significantly following salvage logging operations and did not completely return to prelogging levels by the end of the study period. The largest annual NO3-N export of 13 kg/ha was observed during the first year after harvesting, an increase of approximately 10 kg/ha. Compared to data from other Appalachian Mountain watersheds in North Carolina, West Virginia, and Pennsylvania, calculated N loss for Baldwin Creek was considerably greater. Longer periods of reduced N uptake due to slow revegetation of salvage logged areas, coupled with increased amounts of N available to leaching, could have accounted for the large N losses observed for Baldwin Creek. Salvage logging of dead and dying trees from forested watersheds in this region appears to have the potential to result in much larger N losses than previously reported for harvest of healthy stands.

## INTRODUCTION

The effects of forest harvesting on water quality have been studied extensively since the 1970s, with nitrogen (N) often exhibiting the most significant responses to disturbance compared to other nutrients[1,2,3,4]. Harvesting causes reduced N uptake and increased soil temperature and moisture that result in higher mineralization and nitrification rates[2,4,5]. These changes in N dynamics often cause increased nitrate ($NO_3^-$) leaching below the rooting zone and increased $NO_3^-$ concentrations in streams.

Most studies relating watershed-level nutrient export to forest harvesting have involved commercial removal of apparently healthy stands. However, declining stands can have even greater $NO_3^-$ losses than healthy ones. Durka and colleagues[6] assessed $NO_3^-$ leaching in stream water from declining and nondeclining sites. They found that 16 to 30% of stream water $NO_3^-$ in healthy or slightly declining stands originated directly from the atmosphere, while on more severely declining sites atmospherically deposited $NO_3^-$ was much higher. The authors suggested that, at the former sites, $NO_3^-$ consumption by plants and soil microorganisms was large enough to assimilate most of deposited $NO_3^-$. At declining sites, $NO_3^-$ consumption was much lower, and most of the deposited $NO_3^-$ was exported in stream water. According to Van Miegroet and Johnson[7], natural tree mortality, forest gap formation, and defoliation by insects affect forest soil N dynamics similarly to forest harvesting and result in elevated soil water and stream N levels. Gilliam and Adams[8] examined the effects of forest harvesting on soil N dynamics in an N saturated hardwood forest in the Central Appalachian region. Net N mineralization and nitrification rates were measured and compared between clearcut plots with and without N additions. Both net N mineralization and nitrification were significantly higher at the harvested plots that had N additions.

Hypotheses about N saturation were developed with respect to the capacity of forest soils to retain N[7,9,10]. Soils become N saturated when atmospheric N and internal N mineralization inputs exceed N retention via microbial immobilization and plant uptake. In turn, nitrification and $NO_3^-$ leaching below the rooting zone occur, elevating stream water concentrations of $NO_3^-$ and aluminum (Al). Increased cation leaching from the soil can simultaneously occur and reduce site fertility and increase soil acidity. N saturated soils may cause severe imbalances in element concentrations in foliage, leading to a loss of frost hardiness, and reduced fine-root biomass. Disruptions of tree physiology could diminish forest productivity and contribute to decline and mortality of trees[7,9,10].

Because both forest harvesting and forest decline alter N cycles by increasing N availability (through accelerating N mineralization and nitrification rates) and decreasing vegetative N demand, salvage logging operations likely would significantly affect N cycling, due to the combined effects of harvesting and forest decline. Rapid regeneration of harvested sites is important to minimizing nutrient losses from harvested areas. Since rapid regeneration may not occur on declining sites, nutrient losses from logging of declining sites may be greater in magnitude and duration than from logging sites with vigorous stands.

The objective of this research was to assess N export from the Baldwin Creek watershed in southwestern Pennsylvania, which was partially salvage logged to recover dead and dying hardwoods, primarily northern red oak (*Quercus rubra*). The study involved two steps: (1) determining changes in N export from the disturbed watershed, using stream flow and stream N concentration data and (2) comparing results obtained with data from a nearby control stream (Linn Run) and other studies involving conventionally clearcut catchments. We hypothesized that significant increases in N export would occur following the disturbance on the Baldwin Creek watershed and that salvage logging would induce greater N losses, compared to clearcut watersheds with essentially healthy vegetation at the time of harvest.

## EXPERIMENTAL METHODS/PROCEDURES

### Study Area

The salvage logged and control watersheds (Baldwin Creek and Linn Run, respectively) are located on the Laurel Hill in southwestern Pennsylvania, in the northern portion of the Appalachian Plateau Province. Baldwin Creek and Linn Run are second-order streams on unglaciated, forested watersheds located about 25 km apart. Detailed watershed descriptions are given by DeWalle and others[11]. Stream flow and chemistry data have been collected at continuous monitoring stations on Baldwin Creek and Linn Run from October 1988 to the present. Watershed areas at the continuous monitoring stations are 535 ha for Baldwin Creek and 1000 ha for Linn Run. Mature forests on both watersheds originate from cutting done near the turn of the century.

Laurel Hill lies directly in the path of weather systems that carry acidic emissions from the industrialized Ohio Valley, and the area historically received high amounts of acid deposition[12,13,14]. Much of the area is underlain by acidic sandstone and, as a result, most of the soils are naturally acidic. Demchik and Sharpe[15] suggested that soil acidification could be responsible for observed forest decline in the area. Sweeney[16] observed large losses of base cations from Baldwin Creek and Linn Run and speculated that these losses occurred as a result of atmospheric deposition. Demchik and Sharpe[15] conducted a study of high- and low-mortality red oak plots located in the Laurel Hill region. Soils in the high-mortality plots had lower base cation levels, higher available Al, and lower Ca to Al ratios. Soil liming significantly improved northern red oak growth. Trees on Baldwin Creek watershed were defoliated by gypsy moth for several years prior to salvage logging. This additional stress most likely contributed to the observed decline in northern red oak.

### Soils and Vegetation

In June 2000, we assessed vegetative cover and soil chemistry on Baldwin Creek watershed. Two transects running from the bottom to the top of the watershed were established, and 25 sampling locations (240 m apart) were positioned along the two transects (Fig. 1). At each sampling location, one 4-m diameter plot was established. To assess chemical characteristics of soils, we collected mineral (15- to 20-cm depth) and organic soil samples at each of the 25 sampling locations. Soils were analyzed at the Agricultural Analytical Services Laboratory at Pennsylvania State University, following the recommended soil testing procedures for the northeastern U.S.[17]. Ca to Al ratios were determined using 0.01 *M* $SrCl_2$ extraction[18,19]. $NO_3$-N, $NH_4$-N, and TKN (total kjeldahl N) were determined following the procedures described in Sparks[20]. Soil analyses generally confirmed the presence of acid, infertile soils on the watershed (Table 1). Mineral soil had a mean pH of 4.0, with low base cation levels and a low cation exchange capacity (CEC). Williard and colleagues[21] previously reported that soils on Baldwin Creek and Linn Run have similar N concentrations and similar C to N ratios.

To assess regeneration, we determined percent ground cover for all vegetation within each of the 25 plots and counted the number of seedlings by species. Regeneration was poor, especially at the higher-elevation plots within the watershed. Leaf litter and hayscented fern (*Dennstaedtia punctilobula*) accounted for more than 75% of ground cover, while tree seedlings like sweet birch (*Betula lenta*), red maple (*Acer rubrum*), and northern red oak accounted for less than 15%. The mean number of woody stems per square meter of watershed was remarkably low for all species (<0.03 stems/m$^2$), providing further evidence of poor regeneration. The most common woody species were sassafras (*Sassafras albidum*), red maple, northern red oak, sweet birch, and witch hazel (*Hamamelis virginiana*).

Tree mortality was assessed by examining the ten overstory trees closest to the center of each plot and recording the number of dead trees and the number of trees with >50% crown loss. An average of 40.4% of the trees observed near the 25 plots on the watershed were either dead or had >50% dieback. Tree mortality

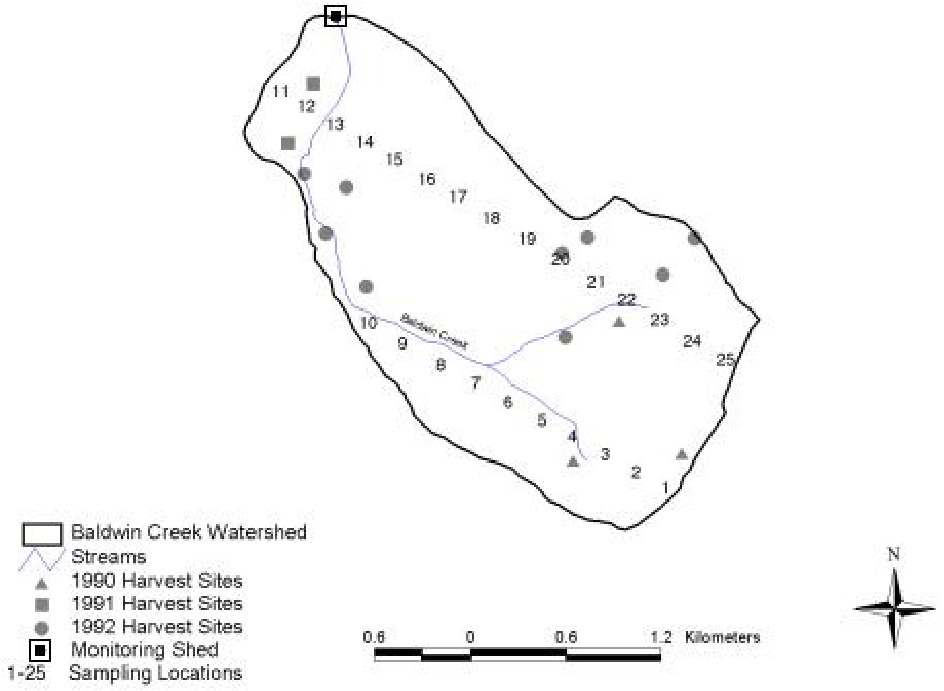

**FIGURE 1.** Approximate location of salvage-logged areas and soil and vegetation sampling plots on the Baldwin Creek watershed.

**TABLE 1**
**Chemical Characteristics of Soils on the Baldwin Creek Watershed**

| | Organic Soil | | Mineral Soil | |
|---|---|---|---|---|
| **Parameter** | **Mean** | **Range** | **Mean** | **Range** |
| pH | 3.8 | 3.4 – 4.2 | 4.0 | 3.5 – 4.6 |
| Acidity (meq/100 g) | 22.5 | 12.6 – 29.7 | 12.7 | 4.1 – 22.4 |
| CEC (meq/100 g) | 18.7 | 15.5 – 21.2 | 12.8 | 4.8 – 16.9 |
| % K Saturation | 1.4 | 0.6 – 2.6 | 0.6 | 0.1 – 1.5 |
| % Mg Saturation | 2.6 | 1.6 – 3.7 | 1.7 | 1.1 – 3.6 |
| % Ca Saturation | 15.8 | 8.6 – 24.7 | 5.7 | 3.1 – 12.1 |
| $NO_3$-N (mg/kg) | 53.6 | 7.6 – 228.5 | 4.5 | 0.83 – 22.4 |
| $NH_4$-N (mg/kg) | 169.2 | 29.8 – 467 | 12.2 | 2.1 – 66.2 |
| TKN (%) | 1.7 | 0.5 – 2.3 | 0.2 | 0.07 – 0.99 |
| Ca:Al molar ratio | 52 | 11.4 – 128.5 | 2.1 | 0.4 – 9.9 |

and decline were also significantly greater on the higher elevation plots.

In response to the extensive decline and mortality of northern red oak on the Baldwin Creek watershed, the Pennsylvania Game Commission conducted extensive clearcutting and thinning during the early 1990s to salvage dead and dying trees. Harvest records were acquired from the Game Commission to document the location, timing, and size of all harvest areas on the watershed (Table 2). Approximately 64% of the watershed was harvested in some way during 1990 to 1992 at the locations shown in Fig. 1.

## Stream Export of N

Hourly stream flow data were collected from Baldwin Creek during 1988 to 1998 using a continuous water level recorder. A total of 443 samples were collected and analyzed for $NO_3^-$ and $NH_4^+$ during the same time period. Sampling frequency for $NO_3$ and $NH_4$ varied considerably during the study period, from hourly to monthly. Detailed descriptions of stream data collection and analysis are available from DeWalle and others[11].

Because of variable chemistry sampling frequencies, the period-weighted method was thought to be appropriate for stream

**TABLE 2**
**Areal Extent of Salvage Logging Operations on the Baldwin Creek Watershed**

| Year | Clearcut Area ha (%) | Selectively Cut Area ha (%) | Total Cut ha (%) |
|---|---|---|---|
| 1990 | 25 (5) | 43 (8) | 68 (13) |
| 1991 | 20 (4) | 0 | 20 (4) |
| 1992 | 181 (34) | 71 (13) | 252 (47) |
| Total | 226 (42) | 114 (21) | 340 (64) |

export calculations[1]. Solute concentrations taken at the beginning and end of an interval were averaged and multiplied by the total stream flow for that period. The following formula was used to compute $NO_3^-$ and $NH_4^+$ fluxes for a desired interval, using an appropriate conversion factor to yield the results in kg/ha:

$$F\ (\text{kg/ha}) = Q\ (\text{m}^3/\text{km}^2) \times C\ (\text{mg/l}) \times 10^{-5} \quad (1)$$

where F is the flux for the period, Q is stream flow for the period, and C is average concentration for the period. Computed $NO_3^-$ and $NH_4^+$ fluxes then were converted to $NO_3$-N and $NH_4$-N and summed to determine monthly and yearly stream export. A similar procedure was used by Sweeney[16].

Mean monthly and mean annual concentrations were calculated as weighted averages by dividing the total export of ionic species for a period by the total stream flow for that period[1]. Two sample t-tests were conducted to compare presalvage logging with postsalvage logging concentration, stream flow, and export values.

Large gaps in stream flow and chemistry data existed for the periods from June 1990 through October 1991 and January 1996 through November 1996, and these periods were not included in the analysis. For three smaller gaps (not exceeding 2 months), stream flow or average concentration values for the period were approximated by averaging the adjacent monthly stream flow and average monthly concentration values at the beginning and end of the period.

The paired watershed method was used to quantify changes in N export from Baldwin Creek, with Linn Run as a reference stream[2]. A calibration equation relating N export from Baldwin Creek to N export from Linn Run was developed for the presalvage logging period. This equation was used to predict N export for Baldwin Creek after salvage logging, and differences between the observed and predicted values were calculated. The difficulty with using the paired watershed approach was that only 1 year (1989) of calibration data was available. However, the 1989 water year was considered representative of the postsalvage logging period in terms of both precipitation and stream flow.

Results for Baldwin Creek are presented on the basis of an October-to-September water year. Thus, the period from October 1, 1988, through September 30, 1989, is the 1989 water year. The 1991 and 1996 water years were not included in the study, since stream flow and chemistry were not monitored during these periods. In addition, because of missing stream flow and chemistry data, the 1990 water year covers only a 9-month period, the 1992 water year covers only an 11-month period, and the1997 water year covers a 10-month period.

Given the data availability constraints, analyses were divided into three intervals. The presalvage logging period included water year 1989, the salvage logging period included the 1990 and 1992 water years, and the postsalvage logging period included water years 1993 to 1998.

Results of the following studies in the eastern U.S., relating watershed-level N export to forest harvesting, were obtained from the literature and compared to results from Baldwin Creek:

- Watershed 2 (clearcut and herbicided) at the Hubbard Brook Experimental Forest in New Hampshire; detailed description and results in Likens and colleagues[22];
- Watershed 7 (clearcut) at the Coweeta Hydrologic Laboratory in North Carolina; detailed description and results in Swank[23];
- Watershed 3 (clearcut) at the Leading Ridge Watershed Research Unit in Pennsylvania; detailed description and results in Lynch and Corbett[24];
- Watershed 3 (clearcut) at the Fernow Experimental Forest in West Virginia; detailed description and results in Aubertin and Patric[25];
- South Haddix watershed (clearcut) at the Fernow Experimental Forest in West Virginia; detailed description and results in Kochenderfer and Edwards[26].

Where possible, the literature $NO_3$-N results from these studies were converted to kg/ha/year. For watershed 2 at the Hubbard Brook Experimental forest, watershed 3 at the Leading Ridge Research Unit, and watershed 3 at the Fernow Experimental Forest, the absolute increases in $NO_3$-N export following clearcutting were not reported. For these watersheds, we calculated the $NO_3$-N export increase following clearcutting as the difference between the reported $NO_3$-N export value for each year after cutting and the mean pretreatment $NO_3$-N export.

## RESULTS AND DISCUSSION

### Trends in Stream Flow, N Concentration and N Export

Stream flow in Baldwin Creek varied considerably over the 10-year study period (Fig. 2A). Stream flow was near normal during the 1989, 1992, 1997, and 1998 water years. Below-average stream flows were observed during 1990 and 1995, when

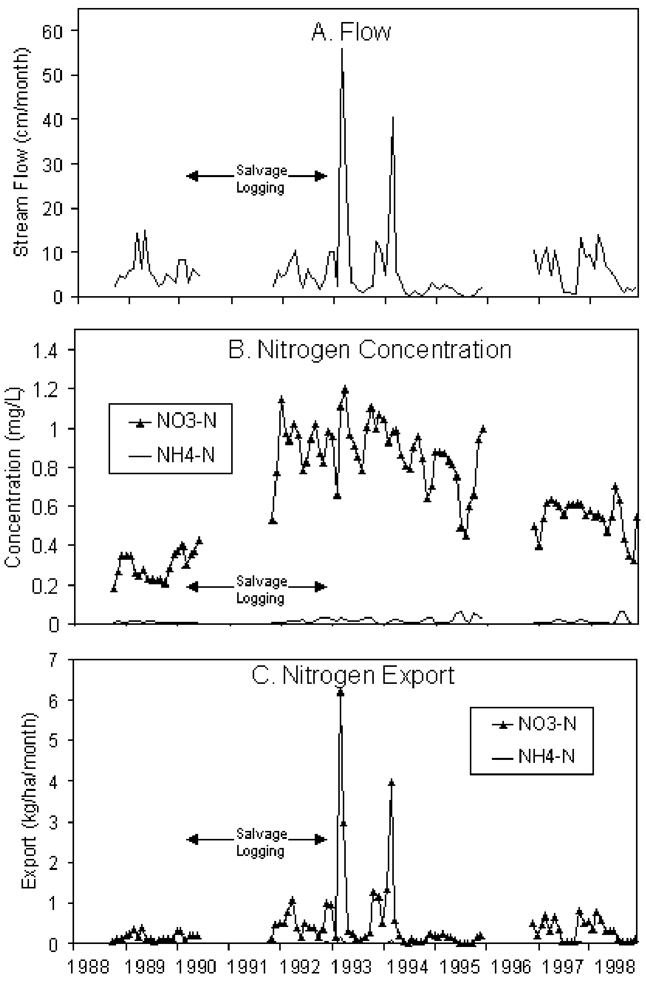

**FIGURE 2.** Mean monthly stream flow (A), stream N concentrations (B), and stream N export (C) for the Baldwin Creek watershed.

droughts occurred. Much above-normal stream flows occurred in both 1993 and 1994, mostly in response to heavy winter snows and spring rainfall (Fig. 2A).

Virtually all of the total annual N export from Baldwin Creek occurred as $NO_3$-N, because stream $NH_4$-N exports were very low (Fig. 2C) and changed little with time (Fig. 2B). $NH_4$-N concentrations in Baldwin Creek were always less than 0.06 mg/l and were often below detection limits. Similar patterns of low $NH_4$-N leaching have been reported elsewhere[22,23,25]. $NH_4^+$ is not very mobile and is not readily leached out of soils, because it can be assimilated by plants and microorganisms, oxidized to $NH_3$ and $NO_3^-$ by nitrifying bacteria (nitrification), adsorbed onto soil colloids, fixed in the interlayer of clays, or reacted with soil organic matter. In contrast, $NO_3^-$ is not adsorbed strongly and is highly soluble; therefore, it is much more mobile and easily leached[10]. It was the primary component of total N and responded markedly to salvage logging. Stream $NO_3$-N concentrations increased considerably after salvage logging and then decreased gradually after October 1993. However, $NO_3$-N concentrations did not return to pretreatment levels by the end of the 10-year period. Similar $NO_3$-N responses have been reported in the literature[22,23,25].

The highest stream flow and N export from Baldwin Creek occurred in the spring and summer months of 1993 and 1994 (Fig. 2). Other studies have demonstrated similar patterns of N loss after tree harvesting[2,22,24]. This is likely due to increased soil temperatures and subsequent biological activity combined with reduced uptake by harvested vegetation. Recent research by Eshleman and others[27] indicated that gypsy moth defoliation could cause elevated N leaching. While extensive gypsy moth defoliation did occur on the Baldwin Creek watershed, the observed increases in N leaching occurred shortly after salvage logging and did not appear to be related to earlier defoliation episodes.

Mean annual stream N concentrations for all years after 1989 were significantly greater than the presalvage logging value (Table 3). However, stream N export increases were not significant in 1990 or 1995. The low N export during 1990 was probably due to a combination of below-average stream flow (Table 3) and the small area (13%) of the watershed that was salvage logged during that year. The greatest amount of logging took place in 1992, and the most significant effects were observed during 1993 and 1994, when both stream flow and N concentrations were high (Table 3). The 1995 water year included a major drought

**TABLE 3**
**Mean Annual N Concentrations, Mean Monthly Discharge, Mean Monthly N Export, Total Annual Discharge, and Total Annual Stream N Export for Baldwin Creek Watershed**

| Year | N conc. (mg/l) | Monthly Stream Discharge (cm/mo) | Monthly N Export (kg/ha/mo) | Total Discharge (cm/year) | Total N Export (kg/ha/year) |
|---|---|---|---|---|---|
| 1989 — Prelogging | 0.27 | 6.31 | 0.18 | 75.75 | 2.13 |
| 1990 — Logging | 0.35[a] | 5.43 | 0.19 | 48.88 | 1.73 |
| 1991 — Logging | | | *Insufficient data* | | |
| 1992 — Logging | 0.91[a] | 5.18 | 0.49[a] | 56.93 | 5.35 |
| 1993 — Postlogging | 0.95[a] | 9.90 | 1.09[a] | 119.16 | 13.00 |
| 1994 — Postlogging | 0.96[a] | 8.10 | 0.81[a] | 96.94 | 9.70 |
| 1995 — Postlogging | 0.75[a] | 1.41 | 0.11 | 16.91 | 1.37 |
| 1996 — Postlogging | | | *Insufficient data* | | |
| 1997 — Postlogging | 0.58[a] | 5.95 | 0.34[a] | 59.53 | 3.41 |
| 1998 — Postlogging | 0.58[a] | 7.04 | 0.40[a] | 84.52 | 4.86 |

[a] Value is significantly greater than the 1989 (presalvage logging) value in the same column (two sample t-test), ($\alpha$ = 0.05).

that produced the lowest stream flow during the study period. Since stream export is predominantly a function of stream flow, even a significant concentration increase, coupled with a decrease in discharge, can result in no increase or even a decrease in stream exports. Annual postlogging stream flow was not significantly different than 1989, even when mean and total discharges increased by more than 50% (Table 3). This is presumably due to the large associated variability in the stream flow data. Mean N concentration and annual N export were still significantly higher then presalvage logging levels at the end of the study in 1997 and 1998 (Table 3).

A linear regression line was fitted to the 1989 monthly $NO_3$-N export data from Baldwin Creek plotted against that from Linn Run (Fig. 3). This regression equation then was used to predict monthly $NO_3$-N exports from Baldwin Creek from 1990 to 1998, had salvage logging not occurred on the watershed. Predicted monthly values were summed by water year. Differences between observed and predicted values represent $NO_3$-N export increases attributable to salvage logging. Confidence limits ($\alpha$ = 0.05) calculated around the predicted monthly values were used to determine whether observed and predicted annual $NO_3$-N exports were significantly different. No significant $NO_3$-N export increases occurred in 1990 and 1995 (Fig. 4). Again, this was likely due to below-normal stream flow during both years and the small amount of salvage logging during 1990. The greatest increases in $NO_3$-N occurred in 1992, 1993, and 1994, immediately following salvage logging activities; however, statistically significant increases were still evident in 1997 and 1998 (Fig. 4).

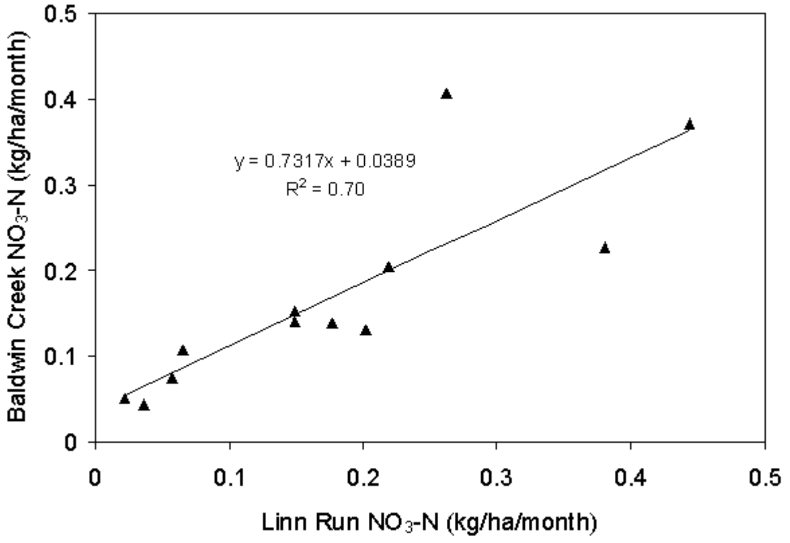

**FIGURE 3.** Presalvage logging relationship between monthly $NO_3$-N stream export from Baldwin Creek and Linn Run. Points represent monthly values from 1989 prior to salvage logging.

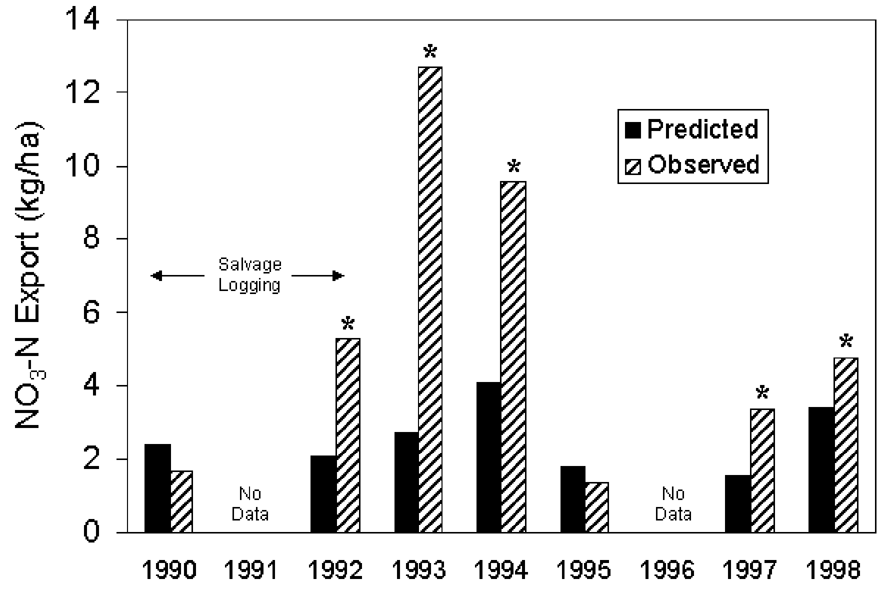

**FIGURE 4.** Predicted and observed $NO_3$-N export from Baldwin Creek during and following salvage logging (paired watershed method). *Note*: An asterisk (*) above the pair of bars indicates that the observed $NO_3$-N export was significantly greater than the predicted value for 50% or more of months of that water year.

## Baldwin Creek's Response Compared to Previous Watershed Studies

Table 4 compares the calculated increases in $NO_3$-N export from Baldwin Creek following salvage logging with the results of the other watershed studies outlined previously. Most of the reviewed studies involved commercial clearcutting of the total watershed area, except for Leading Ridge where only 43% of the total watershed area was clearcut. Salvage logging activities on Baldwin Creek included clearcutting of about 42% of the total watershed area and selective cutting of about 22% of the total area (Table 2). Martin and Pierce[28] provided evidence that clearcutting less than the entire watershed area reduced the magnitude of stream $NO_3$-N concentration increases, compared to clearcutting the total watershed area. They suggested that clearcutting the entire drainage area caused the greatest nutrient export. If the entire area of Baldwin Creek watershed were clearcut, annual $NO_3$-N exports would likely have increased more than was observed. Therefore, to compare Baldwin Creek to other catchments with watershed-wide clearcuts, the $NO_3$-N export values were multiplied by a simple area-based weighting factor to approximate Baldwin Creek's response if the entire watershed had been clearcut. Because salvage logging on Baldwin Creek watershed included both clearcut and selectively cut areas, and we had no means of determining how the effects of clearcutting differed from the effects of selective cutting, we considered two extreme scenarios and used two different weighting factors. If selective cutting and clearcutting were assumed to have comparable effects on $NO_3$-N export, then all of the salvage logged areas, or approximately 64% of Baldwin Creek watershed area, could be considered clearcut. In this case, the appropriate area-based weighting factor for the entire watershed would be 1.56. On the other hand, if selective cutting were assumed to have a negligible effect, then only 42% of Baldwin Creek watershed would have an effect on $NO_3$-N export. In this case the appropriate area-based weighting factor for the entire watershed would be 2.38. Thus, in Table 4, Baldwin Creek × 1.56 represents a more conservative estimate of Baldwin Creek's response to salvage logging, while Baldwin Creek × 2.38 represents a less conservative estimate.

N export from Baldwin Creek was much higher than all other watersheds, with the exception of Hubbard Brook (Table 4). The Hubbard Brook watershed is unique in this comparison, because herbicides were repeatedly applied to suppress regrowth after cutting. In comparison to watersheds without herbicide applications, Baldwin Creek N export was four to 250 times greater, due to cutting and continual tree decline. We estimated that the Baldwin Creek watershed still had 40% tree mortality in year 2000 — 8 years after major salvage logging had occurred. Greater N export from Baldwin Creek may have occurred because salvage logging further reduced N demand, which was already low because of the significant tree decline[6]. At the same time, forest decline may have resulted in greater availability of organic matter (leaf and limb litter from declining trees), higher soil moisture content (due to reduced water uptake by declining trees), and higher soil temperatures (due to thinner canopy of declining trees). These factors are favorable to N mineralization and nitrification and could have increased amounts of N available for leaching. Baldwin Creek is located in an area of historically high acid deposition, and high atmospheric inputs of N may also have contributed to the high N export observed[12,13,14].

Marks and Bormann[29] suggested that quick regeneration prevented dramatic nutrient losses from clearcut areas. Vegetation assessment on Baldwin Creek watershed revealed unsuccessful regeneration across the watershed. Unsuccessful woody regeneration may have prolonged the period of reduced N uptake by vegetation on Baldwin Creek watershed, compared to other watersheds where rapid regeneration was observed after

**TABLE 4**
**Increases in $NO_3$-N Export (kg/ha) Following Commercial Harvesting of Several Watersheds, in Comparison to Baldwin Creek**

| | Years after Harvest | | | | | |
|---|---|---|---|---|---|---|
| **Watershed** | **1** | **2** | **3** | **4** | **5** | **6** |
| Hubbard Brook | 102.7 | 145.0 | ** | ** | ** | ** |
| Leading Ridge[a] | 0.04 | 0.02 | 0.05 | ** | 0 | 0 |
| Fernow | 2.4 | 1.61 | ** | ** | ** | ** |
| Coweeta | 0.26 | 1.12 | 1.27 | 0.25 | 0.28 | ** |
| South Haddix | 1.77 | 2.69 | 2.75 | ** | ** | ** |
| Baldwin Creek[a] | 10.0 | 5.48 | 0[b] | ** | 1.81 | 1.36 |
| Baldwin Creek × 1.56 | 15.6 | 8.55 | 0[b] | ** | 2.82 | 2.12 |
| Baldwin Creek × 2.38 | 23.8 | 13.04 | 0[b] | ** | 4.31 | 3.24 |

*Note:* ** = no data available.

[a] Less than 100% of the total watershed area was harvested.

[b] Severely reduced stream flow due to drought.

cutting. Prolonged reduced N uptake could partially explain why stream N concentration and export from Baldwin Creek were slow to return to presalvage logging conditions (Table 3).

## CONCLUSIONS

$NO_3$-N export from Baldwin Creek increased significantly following salvage logging operations. Annual $NO_3$-N export was highest during the first year after harvesting (1993), totaling 13 kg/ha. During that year, $NO_3$-N export increased approximately 10 kg/ha over the predicted $NO_3$-N export. At the end of the study period, N concentrations and exports remained higher than presalvage logging values. Compared to other watersheds in North Carolina, Pennsylvania, and West Virginia, N losses from Baldwin Creek following harvesting were considerably greater.

These analyses support the hypotheses that significant increases in N export occurred following harvesting on Baldwin Creek watershed and that salvage logging to remove dead and dying trees induced greater N losses in comparison to cutting of basins with apparently healthy vegetation. We believe the extended reduction of N uptake due to slow revegetation in salvage logged areas, coupled with increased amounts of N available to leaching, was responsible for the larger magnitude and duration of N losses from the Baldwin Creek watershed. The results of this study infer that watersheds experiencing forest decline will have N losses several times greater than previously reported for harvesting activities on more vigorously growing trees.

Results from this study may have important implications for management of watersheds with declining forests. Our results suggest that the potential water quality impacts of salvage logging should be considered more carefully by forest managers. In some cases, salvage logging of dead and dying hardwoods may not be advisable. More research is needed to better quantify the edaphic characteristics of sites where salvage logging may result in excessive N export.

## ACKNOWLEDGMENTS

Although the research described in this paper has been funded in part by the U.S. Environmental Protection Agency agreement CR-8818735-01-0 to The Pennsylvania State University, it has not been subjected to the Agency's review and, therefore, does not necessarily reflect the views of the Agency, and no official endorsement should be inferred. This research was also funded in part by the U.S. Geological Survey. The authors would like to thank Pamela J. Edwards and James A. Lynch for providing reviews of the original manuscript.

## References

1. Likens, G.E., Bormann, F.H., Pierce, R.S., Eaton, J.S., and Johnson, N.M. (1977) *Biochemistry of a Forested Ecosystem*. Springer-Verlag, New York. 146 p.
2. Brooks, K.N., Ffolliott, P.F., Gregersen, H.M., and DeBano, L.F. (1997) *Hydrology and the Management of Watersheds*. Iowa State University Press, Ames. 502 p.
3. Hornbeck, J.W., Martin, C.W., Pierce, R.S., Bormann, F.H., Likens, G.E., and Eaton, J.S. (1987) The Northern Hardwood Ecosystem: Ten Years of Recovery from Clearcutting. USDA Forest Service, Northeastern Forest Experiment Station, Report NE-RP-596. U.S. Department of Agriculture Forest Service, Northeastern Forest Experiment Station, Morgantown, WV. 30 p.
4. Pardo, L.H., Driscoll, C.T., and Likens, G.E. (1995) Patterns of nitrate loss from a chronosequence of clear-cut watersheds. *Water Air Soil Pollut.* **85**, 1659–1664.
5. Huttl, R.F. and Schaaf, W. (1995) Nutrient supply of forest soils in relation to management and site history. *Plant Soil* **168–169**, 31–41.
6. Durka, W., Schulze, E.D., Gebauer, G., and Voerkelius, S. (1994) Effects of forest decline on uptake and leaching of deposited nitrate determined from $^{15}N$ and $^{18}O$ measurements. *Nature* **372**, 765–767.

7. Van Miegroet, H. and Johnson, D.W. (1993) Nitrate dynamics in forest soils. In *Nitrate: Processes, Patterns and Management.* John Wiley & Sons, New York. pp. 75–88.
8. Gilliam, F.S. and Adams, M.B. (1999) Effects of harvesting on soil nitrogen dynamics in a N saturated hardwood forest. In *Proc. 12th Central Hardwood Forest Conference; 1999. February 28, March 1–2; Lexington, KY.* Gen. Tech. Rep. SRS-24. U.S. Department of Agriculture, Forest Service, Southern Research Station, Asheville, NC. pp. 29–36.
9. Aber, J.D., Knute, J.K., Nadelhoffer, J., Steudler, P., and Melillo, J.M. (1989) Nitrogen saturation in northern forest ecosystems. *BioScience* **39**, 378–386.
10. Dail, B., Chorover, J., and Davidson, E. (1999) Nitrogen cycling in forest ecosystems: impact of acidic deposition. In *The Effects of Acid Deposition on Pennsylvania's Forests.* Drohan, J.R. and Sharpe, W.E., Eds. Environmental Resources Research Institute, University Park, PA. pp. 29–44.
11. DeWalle, D.R., Swistock, B.R., Dow, C.L., Sharpe, W.E., and Carline, R.F. (1992) *Episodic Response Project — Northern Appalachian Plateau: Site Description and Methodology*. U.S. Environmental Protection Agency, Environmental Research Laboratory, Corvalis, OR. 55 p.
12. Kimmel, W.K. (1999) Macroinvertebrate community responses to episodic stream acidification on the Laurel Hill in southwestern Pennsylvania. In *The Effects of Acidic Deposition on Aquatic Ecosystems in Pennsylvania.* Drohan, J.R. and Sharpe, W.E., Eds. Environmental Resources Research Institute, University Park, PA. pp. 17–22.
13. DeWalle, D.R., Sharpe, W.E., and Edwards, P.J. (1988) Biogeochemistry of two Appalachian deciduous forest sites in relation to episodic stream acidification. *Water Air Soil Pollut.* **40**, 143–156.
14. Lynch, J.A., Grimm, J.W., and Horner, K.S. (1997) Atmospheric Deposition: Spatial and Temporal Variations in Pennsylvania — 1996. Publication No. ER9709, Environmental Resources Research Institute, University Park, PA.
15. Demchik, M.C. and Sharpe, W.E. (1999) The effects of soil acidification and liming on northern red oak decline in southwestern Pennsylvania. In *The Effects of Acid Deposition on Pennsylvania's Forests.* Drohan, J.R. and Sharpe, W.E., Eds. Environmental Resources Research Institute, University Park, PA. pp. 161–170.
16. Sweeney, J.S. (1998) Chemical Mass Balances for Five Appalachian Plateau Watersheds in Pennsylvania (1988 – 1995) [M.S. Thesis] Pennsylvania State University, University Park, PA. 95 p.
17. Sims, J.T. and Wolf, A., Eds. (1995) Recommended Soil Testing Procedures for the Northeastern United States. Northeastern Regional Bulletin #493. Agricultural Experimental Station, University of Delaware, Newark.
18. Joslin, J.D. and Wolfe, M.H. (1988) Responses of red spruce seedlings to changes in soil aluminum in six amended forest soil horizons. *Can. J. For. Res.* **18**, 1614–1623.
19. Joslin, J.D. and Wolfe, M.H. (1989) Aluminum effects on northern red oak seedling growth in six forest soil horizons. *Soil Sci. Soc. Am. J.* **53**, 274–281.
20. Sparks, D.L., Ed. (1996) *Methods of Soil Analysis, Part 3: Chemical Methods.* Soil Science Society of America Book Series Number 5. American Society of Agronomy, Madison, WI.
21. Williard, K.W., DeWalle, D.R., Edwards, P.J., and Schnabel, R.R. (1997) Indicators of nitrate export from forested watersheds of the mid-Appalachians, United States of America. *Glob. Biogeochem. Cy.* **11**, 649–656.
22. Likens, G.E., Bormann, F.H., Johnson N.M., Fisher, D.W., and Pierce, R.S. (1970) Effects of forest cutting and herbicide treatment on nutrient budgets in the Hubbard Brook watershed ecoysystem. *Ecol. Monogr.* **40**, 27–24.
23. Swank, W.T. (1988) Stream chemistry responses to disturbances. *In Forest Hydrology and Ecology at Coweeta.* Springer-Verlag, New York. pp. 339–357
24. Lynch, J.A. and Corbett, E.C. (1991) Long-term implications of forest harvesting on nutrient cycling in central hardwood forests. In *8th Central Hardwood Forest Conference.* General Technical Report NE – 148. McCormick, L.H. and Gottschalk, K.W., Eds. U.S. Department of Agriculture, Randor, PA. pp. 500–518.
25. Aubertin, G.M. and Patric, J.H. (1974) Water quality after clearcutting a small watershed in West Virginia. *J. Environ. Qual.* **3**, 243–249.
26. Kochenderfer, J.N. and Edwards, P.J. (1990) Effectiveness of three streamside management practices in the central Appalachians. In *Proc. Sixth Biennial Southern Silvicultural Research Conference, Vol. 1*. Southeastern Experimental Station, Asheville, NC. pp. 688–700.
27. Eshleman, K.N., Morgan II, R.P., Webb, J.R., Deviney, F.A., and Galloway, J.N. (1998) Temporal patterns of nitrogen leakage from mid-Appalachian forested watersheds: role of insect defoliation. *Water Resour. Res.* **34**, 2005–2116.
28. Martin, C.W. and Pierce, R.S. (1980) Clearcutting patterns affect nitrate and calcium in streams of New Hampshire. *J. For.* May 1980, 268–272.
29. Marks, P.L. and Bormann, F.H. (1972) Revegetation following forest cutting: mechanisms for return to steady-state nutrient cycling. *Science* **176**, 914–916.

| | | |
|---|---|---|
| Received: | July | 13, 2001 |
| Revised: | October | 8, 2001 |
| Accepted: | October | 10, 2001 |
| Published: | November | 9, 2001 |
